# The dynamic conformational landscape of γ-secretase

**DOI:** 10.1242/jcs.164384

**Published:** 2015-02-01

**Authors:** Nadav Elad, Bart De Strooper, Sam Lismont, Wim Hagen, Sarah Veugelen, Muriel Arimon, Katrien Horré, Oksana Berezovska, Carsten Sachse, Lucía Chávez-Gutiérrez

**Affiliations:** 1 VIB Center for the Biology of Disease, 3000 Leuven, Belgium; 2 Center of Human Genetics, University Hospitals Leuven & Department of Human Genetics, KU Leuven, and Leuven Research Institute for Neuroscience and Disease (LIND), 3000 Leuven, Belgium; 3 UCL Institute of Neurology, Queen Square, London WC1N 3BG, UK; 4 European Molecular Biology Laboratory, Structural and Computational Biology Unit, Meyerhofstrasse1, 69117 Heidelberg, Germany; 5 Department of Neurology, Massachusetts General Hospital, Harvard Medical School, Boston, MA 02114, USA

**Keywords:** γ-secretase, Alzheimer disease, Conformational landscape, Regulated intramembrane proteolysis, Single particle electron microscopy

## Abstract

The structure and function of the γ-secretase proteases are of great interest because of their crucial roles in cellular and disease processes. We established a novel purification protocol for the γ-secretase complex that involves a conformation- and complex-specific nanobody, yielding highly pure and active enzyme. Using single particle electron microscopy, we analyzed the γ-secretase structure and its conformational variability. Under steady-state conditions, the complex adopts three major conformations, which differ in overall compactness and relative position of the nicastrin ectodomain. Occupancy of the active or substrate-binding sites by inhibitors differentially stabilizes subpopulations of particles with compact conformations, whereas a mutation linked to familial Alzheimer disease results in enrichment of extended-conformation complexes with increased flexibility. Our study presents the γ-secretase complex as a dynamic population of interconverting conformations, involving rearrangements at the nanometer scale and a high level of structural interdependence between subunits. The fact that protease inhibition or clinical mutations, which affect amyloid β (Aβ) generation, enrich for particular subpopulations of conformers indicates the functional relevance of the observed dynamic changes, which are likely to be instrumental for highly allosteric behavior of the enzyme.

## INTRODUCTION

γ-secretase complexes have the remarkable ability to cleave integral membrane proteins within the lipid bilayer. Proteolytic products provide signals to the extracellular and intracellular environment, coordinating physiological and pathological processes at both sides of the cell membrane (De Strooper and Annaert, 2010; Selkoe and Wolfe, 2007). Best studied are the roles of γ-secretases in the processing of the amyloid precursor protein (APP) and the secretion of the neurotoxic amyloid β (Aβ) peptides in the context of Alzheimer disease. However, other substrates, including Notch, N-cadherin and ErbB4, link γ-secretase activities to development, cancer and immunity (De Strooper and Annaert, 2010; Selkoe and Wolfe, 2007).

**Fig. 1.**
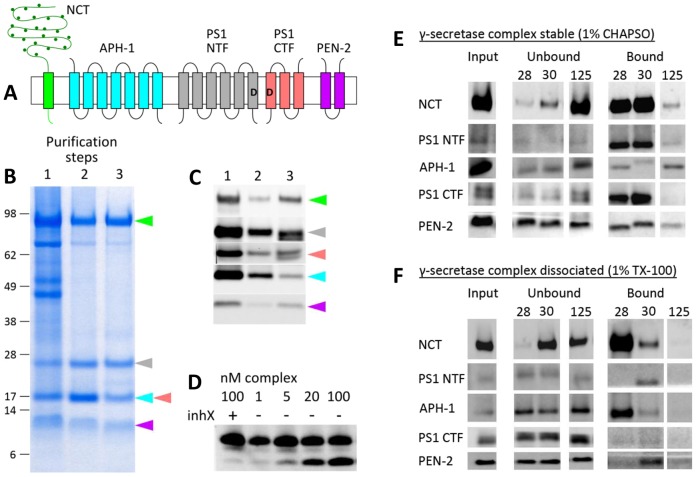
**γ-secretase purification using complex-specific Nb30.** (A) Membrane topology of the five γ-secretase subunits. Subunits include presenilin 1 N-terminal fragment (PS1-NTF) and C-terminal fragment (PS1-CTF), which contain the catalytic aspartates (′D′). Nicastrin (NCT) is a type 1 integral membrane glycoprotein of 130 kDa, comprising a large ectodomain (ECD); anterior pharynx defective 1 (APH-1) is a 30-kDa protein with seven TMDs; and presenilin enhancer 2 (PEN-2) is a 12-kDa hairpin-like protein with two TMDs. (B,C) Coomassie-stained SDS-PAGE and western blotting following the three purification steps: (1) anti-GFP nanobody affinity chromatography and elution by protease cleavage, (2) binding to complex-specific Nb30–His and IMAC, (3) size-exclusion chromatography. Colored arrowheads mark the different subunits according to the color scheme in A. (D) Activity assay of the purified γ-secretase as measured by APP intracellular domain (AICD) generation from APP C99x3FLAG substrate. Increasing amounts of AICD product (lower lanes) are seen with increasing concentrations of purified γ-secretase (lanes 2–5). Additionally, purified γ-secretase is inhibited specifically by transition-state analog inhX (lane 1). (E,F) Nb30 immunoprecipitates only the assembled γ-secretase complex, whereas it fails to bind γ-secretase under dissociating conditions (TX-100). Hi5 insect cells overexpressing γ-secretase complexes were solubilized in either 1% CHAPSO (E) or 1% Triton X-100 (F) and used in immunoprecipitation experiments with Nb28, Nb30 and Nb125. The active γ-secretase complex is stable in 1% CHAPSO, whereas 1% Triton X-100 dissociates the complex. Shown are SDS-PAGE and western blotting analyses of input, unbound and bound fractions. Immunostaining for γ-secretase subunits indicates that Nb30 binds γ-secretase when the complex is stable (1% CHAPSO, E), but does not bind the individual subunits (1% Triton X-100, F). In contrast, Nb28 binds both the γ-secretase complex and the dissociated NCT subunit. Nb125 is a non-interacting γ-secretase nanobody used as negative control.

Presenilin 1 (PS1), nicastrin (NCT), presenilin enhancer 2 (PEN-2) and anterior pharynx defective 1A (APH-1A) assemble in a tetrameric complex (Fig. 1A) (De Strooper, 2003) and PS1 autoproteolysis results in an active pentameric γ-secretase (Thinakaran et al., 1996), in which the catalytic center is structured at the interface between the N-terminal and the C-terminal fragments of PS1 (NTF and CTF respectively) (Esler et al., 2000; Li et al., 2013; Li et al., 2000; Wolfe et al., 1999) and connected to the intracellular aqueous environment (Sato et al., 2006; Tolia et al., 2006). NCT, a type 1 integral membrane glycoprotein, plays a crucial role in complex maturation and stabilization (Chávez-Gutiérrez et al., 2008) and might be involved in substrate binding (Shah et al., 2005; Zhang et al., 2012), although this is debated (Chávez-Gutiérrez et al., 2008). The recent crystal structure of the NCT ectodomain (ECD) shows a two-lobed architecture with an interface of van der Waals and hydrogen bonds between the lobes (Xie et al., 2014). APH-1 and PEN-2 contribute seven and two transmembrane domains (TMDs) to the protease complex, respectively, and their roles are poorly understood. The first high-resolution (4.5 Å) structure of γ-secretase was very recently obtained using single particle electron microscopy (Lu et al., 2014). The model presents the NCT ectodomain and traces the 19 TMDs organized in a horseshoe-shaped membrane core. The flexible loops connecting the TMDs and the hydrophilic N- and C-termini of PS1 and PEN-2 were not visualized owing to their high flexibility. Although it represents a major step towards the molecular understanding of γ-secretase mechanisms, additional functional-structural information on the complex is needed to understand how γ-secretase cleaves different substrates and how Alzheimer-causing mutations affect its activity.

γ-secretase is a highly regulated allosteric enzyme with at least two conformations under equilibrium. Differential subunit composition (Serneels et al., 2009), aging-related post-translational modifications caused by oxidative stress (Guix et al., 2012; Wahlster et al., 2013), pathogenic mutations in PS1 (Berezovska et al., 2005) and interaction with substrates or γ-secretase modulators (GSMs) (Uemura et al., 2010) all shift γ-secretase conformational equilibrium and regulate protease activity. With the exception of Li and colleagues (Li et al., 2014), previous single particle electron microscopy studies reported individual structures providing static views of the complex (Lazarov et al., 2006; Li et al., 2014; Lu et al., 2014; Ogura et al., 2006; Osenkowski et al., 2009; Renzi et al., 2011). Li and colleagues (Li et al., 2014) reported differences in the relative position of the NCT ECD in the presence or absence of a non-active-site inhibitor (compound E), providing the first glimpse of structural changes triggered by a non-active-site inhibitor. However, the study focuses on the characterization of single conformations, rather than on the distribution of (multiple) conformations under varying conditions (the ‘allosteric ensemble’).

Based on the highly allosteric behavior of γ-secretase, we postulate that this multimeric complex adopts multiple conformations under steady-state conditions and that ligand (inhibitor, substrate, allosteric modulators) binding or presence of a pathological mutation (mutant PS1) in its active site alters the equilibrium between conformers. In this view, the catalytic activity of γ-secretase is defined by the ‘average activity’ of a set of co-existing conformers, the equilibrium of which depends on presence of ligands, mutations, lipids etc. To test our hypothesis, we purified γ-secretase using a complex-specific nanobody and analyzed the resulting pure and active γ-secretase by single-particle electron microscopy. We show that the wild-type γ-secretase complex exists as an ensemble of at least three distinct major conformations (compact, intermediate and extended), which are in dynamic equilibrium. Inhibition, substrate binding or the PS1 familial Alzheimer disease (FAD)-causing mutation (delta exon 9) remodel the conformational landscape of the protease by enriching specific subpopulations that already pre-exist in the ligand-free state.

## RESULTS

### A novel purification protocol based on an anti-γ-secretase complex-specific nanobody results in highly pure and active enzyme

**Fig. 2.**
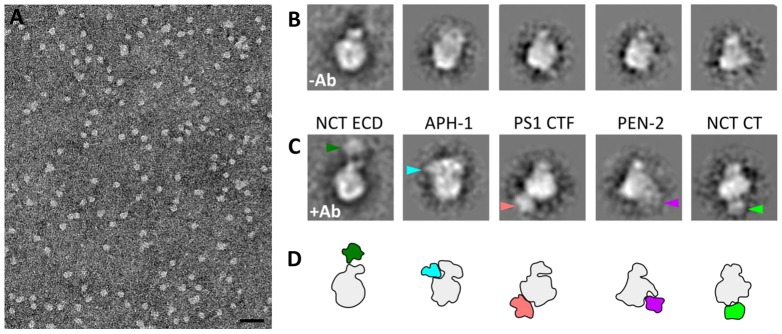
**Electron microscopy characterization of purified γ-secretase complex.** (A) Representative micrograph of the purified γ-secretase. Scale bar: 50 nm. (B,C) Class averages of free (B) and antibody-labeled (C) γ-secretase showing corresponding orientations. The extra density resulting from the bound antibody is indicated by an arrowhead for each of the different antibodies in C. The density of the anchored antibody is enhanced in class averages, whereas other domains of the antibody, which move independently, are averaged out. (D) Outlines of the densities shown in B, γ-secretase is in gray and the antibody densities are shown in color. Additional class averages are shown in supplementary material Fig. S1.

We expressed γ-secretase in insect cells using a baculovirus expression system. We used one-step-purified γ-secretase complex (Fig. 1B, lane 1; Fig. 1C, lane 1) (Acx et al., 2014) to generate single chain camelid anti-γ-secretase nanobodies (Pardon et al., 2014). We identified the conformational nanobody Nb30, which immunoprecipitates γ-secretase in 1% CHAPSO, a condition that preserves the integrity and activity of the complex, but fails to immunoprecipitate any of the γ-secretase subunits in 1% Triton X-100 (TX-100) (complex-dissociating conditions) (Capell et al., 1998) (Fig. 1E,F). Nb30 does not affect γ-secretase activity in an *in vitro* activity assay (data not shown). Therefore, it is unlikely that binding of Nb30 changes γ-secretase conformation, although we cannot reject this possibility. We then incorporated Nb30 in the purification of active γ-secretase (see Materials and Methods). The protocol yields highly pure and active untagged γ-secretase complex (Fig. 1B, lane 2; Fig. 1C, lane 2). We also screened for detergents that keep γ-secretase active and stable. Gel filtration to exchange CHAPSO to lauryl maltose-neopentyl glycol (LMNG) results in active (Fig. 1D) and stable γ-secretase complex. A typical electron microscopy micrograph of negatively stained particles (Fig. 2A) shows that this purification procedure results in monodisperse particles of the expected size.

In order to verify the integrity of the complex, we performed antibody labeling with antibodies against NCT ECD, NCT C-terminus (NCT CT), PS1 CTF, APH-1 and PEN-2, imaged the bound complexes by electron microscopy and performed two-dimensional (2D) analysis. Class averages of antibody-bound enzyme and free γ-secretase were aligned, enabling identification of the bound antibody as an extra density protruding from the γ-secretase projection (Fig. 2B–D; more examples are shown in supplementary material Fig. S1). By analyzing the 2D class averages, we identify the extended density as the NCT ECD (Fig. 2C,D, indicated in dark green). As expected, NCT CT antibodies (light green) were found at an opposing position to the NCT ECD antibodies. In the membrane core, antibodies against the APH-1 CT domain were localized directly beneath the NCT ECD antibodies and the C-terminal part of PS1-CTF antibodies close to the APH-1 epitope, but were distant from the NCT ECD antibodies. PEN-2 antibodies localize at a distant position from the extended domain. Notably, all antibodies bound at a 1∶1 stoichiometric ratio, confirming the integrity of the complex.

### Wild-type γ-secretase complex exists as an ensemble of different conformers

**Fig. 3.**
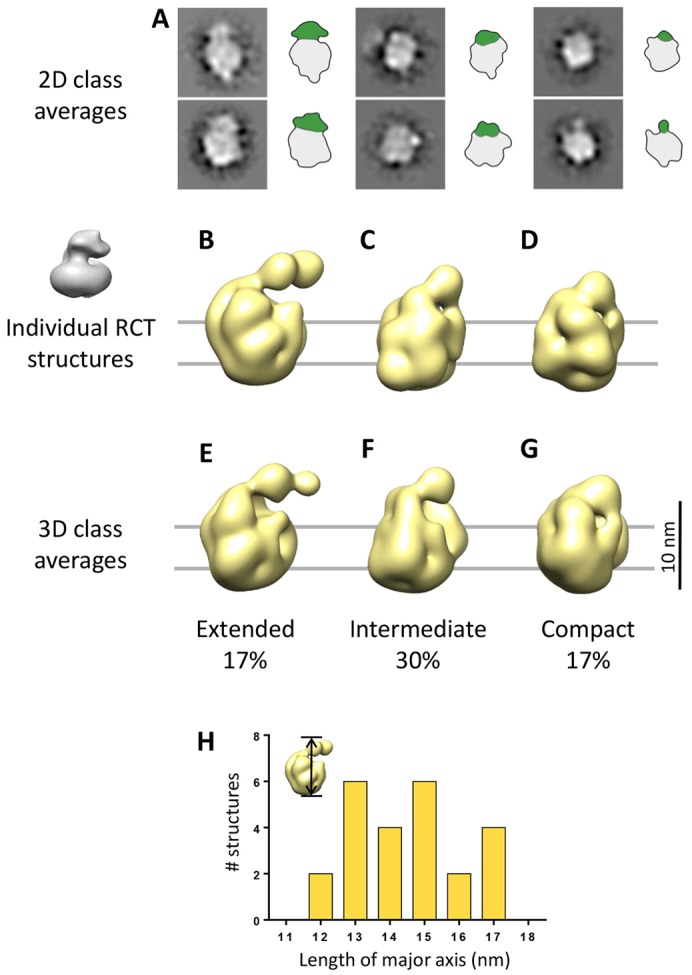
**Variable conformation of wild-type γ-secretase.** (A) Representative class averages used for 3D reconstruction by the RCT method. Outlines of the densities are shown next to class averages. The membrane core is shown in gray and the NCT ECD is in green. Separation into domain is based on the reconstructed 3D maps. (B–D) Representative structures from the different 3D classes – extended, intermediate and compact. Structures are displayed from the side (viewing into the membrane plane). The cryoelectron microscopy structure from Lu et al., 2014 filtered to 30 Å is shown in the inset in gray for comparison. (E–G) 3D class averages calculated by MSA-based hierarchical ascendant 3D classification. The proportion of aligned RCT structures represented by each class is indicated. (H) Length distribution of the γ-secretase structures based on measurements along their major axis.

We investigated the architecture of the γ-secretase complex by single particle electron microscopy. Datasets are *a priori* low-pass filtered to 25 Å in order to avoid any bias of the alignment from noise. Electron microscopy images were analyzed in 2D using reference-free alignment and classification. The class averages show variability in the membrane core and in the relative position of the extended domain density (Fig. 3A). Because variability in 2D projections can result from different conformations or different orientations, multiple initial three-dimensional (3D) maps were reconstructed using the random conical tilt (RCT) method (Radermacher et al., 1987), resulting in 3D structures with resolutions of 23–25 Å (Fig. 3B–D; supplementary material Fig. S2A–F). From a total of 30 3D classes, we found three different structural states – extended, intermediate and compact – which account for 17%, 30% and 17% of the aligned structures, respectively (Fig. 3E–G). Classes differ in overall compactness of the complex, mainly seen as a large hinge rotation of the NCT ECD towards the membrane core. We obtained the same three significant structures using unsupervised 3D classification with RELION software (Scheres, 2012) (supplementary material Fig. S2G), confirming both the overall architecture of γ-secretase structures and the proposed conformational heterogeneity in the preparation. The compact structure resembles previously published electron microscopy structures (Li et al., 2014; Lu et al., 2014), including a membrane core, surrounded by a detergent belt and the NCT ECD. Differences were found in the detergent belt, which appeared to be rougher and thicker in our structures. This can be attributed to the different detergents used in addition to the negative stain method. However, we noted that our obtained structures vary considerably in dimensions and domain architecture, indicating that the complex fluctuates between different conformations under steady-state conditions.

We defined the length of the major axis as a good and simple indication of the population diversity (Fig. 3H). Indeed, a dramatic 30% (5 nm) difference between the compact and extended structures is observed along this axis. Extended, intermediate and compact are 16–17 nm, 14–15 nm and 12–13 nm long, respectively. Importantly, below, we provide evidence that, similarly, large differences in the conformation of the complex are observed under physiological conditions in the cell membrane (Figs 5, 6).

### Inhibitor binding to the γ-secretase complex shifts its conformational equilibrium

**Fig. 4.**
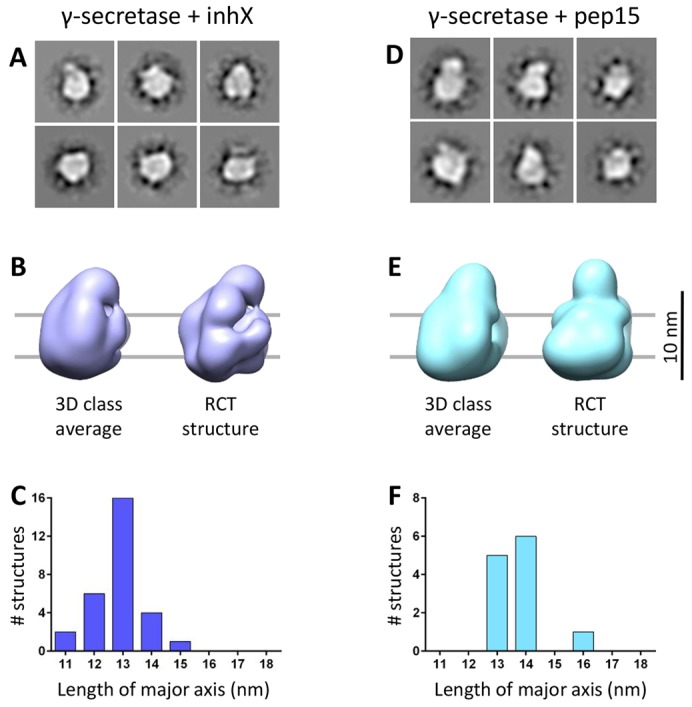
**Differential stabilization of the compact conformation induced by inhX and pep15.** (A,D) Representative class averages from the γ-secretase–inhX and γ-secretase–pep15 datasets, respectively. (B,E) 3D averages and representative RCT structures from the γ-secretase–inhX and γ-secretase–pep15 datasets, respectively, displayed from the side as the untreated γ-secretase structures (Fig. 3B–G). (C,F) Length distribution of the different γ-secretase–inhX and γ-secretase–pep15 structures, respectively, based on measurement along their major axis.

We hypothesized that the equilibrium between the different conformations observed in our experiments (defines protease activity at the macromolecular level) is shifted upon ligand binding to the protease. We tested this by evaluating the shifts in the equilibrium between the different conformers induced by binding of inhibitors to the protease. The transition-state analog inhibitor X (inhX, L-685,458) and inhibitor peptide15 (pep15) bind to the catalytic site and the initial substrate site, respectively. Inhibitor binding, according to our hypothesis, will stabilize particular conformations and thus shift the conformational equilibrium. Indeed, class averages of the γ-secretase–inhX complex (Fig. 4A; supplementary material Fig. S3B) appear to be rounded and less variable at the NCT ECD density when compared with those of untreated γ-secretase (Fig. 3A). Accordingly, inhX narrows the distribution and shifts the mean length of the major axis from 15.3 nm (untreated, Fig. 3H) to 12.8 nm (Fig. 4B,C; supplementary material Fig. S3A,B), indicating that inhibitor-bound γ-secretase is more compact and less structurally heterogeneous. The reduced structural heterogeneity observed in the inhX-treated particles enabled us to validate the overall correctness of the structure by tilt-pair analysis (Rosenthal and Henderson, 2003) (supplementary material Fig. S4).

Next, we used a γ-secretase inhibitor pep15, which is a helical peptide that binds to the putative initial substrate-binding site in the complex (Das et al., 2003). Similar to inhX, pep15 enriches for a compact conformation (Fig. 4D–F; supplementary material Fig. S3C,D), but the NCT ECD is positioned at the edge of the membrane core in the majority of the structures (Fig. 4E). Accordingly, the mean of the length of the major axis is 13.7 nm (Fig. 4F).

**Fig. 5.**
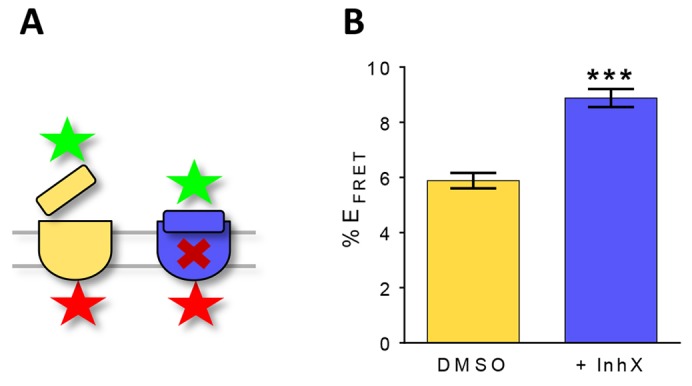
**InhX-induced NCT ECD movement observed in HEK293 cells using FRET-FLIM.** (A) The experimental setup is illustrated. FRET-FLIM was used to monitor the distance between the γ-secretase NCT ECD and NCT CT in HEK293 cells using antibodies carrying donor (green star; rabbit-anti-NCT ECD and donkey anti-rabbit-IgG conjugated to Alexa Fluor 488 as secondary) and acceptor (red star; mouse anti-NCT 9c3 and goat anti-mouse-IgG conjugated to Alexa Fluor 555 as secondary) fluorophores. (B) FRET efficiency was considerably higher following inhX treatment, indicating that the NCT ECD moved closer to the NCT CT on the cytosolic side. Data show the mean±s.e.m.; ***P = 0.0003 (unpaired Student's *t*-test).

We asked whether the large conformational changes we observe in our *in vitro* system could also be observed in γ-secretase complexes in their native state, when embedded in the cell membrane. We therefore studied the effect of inhX on the conformation of γ-secretase using fluorescence-lifetime imaging microscopy (FLIM) on intact cells. We hypothesized that treatment with inhX would increase the proximity of the NCT ECD to the membrane core and therefore to its intracellular domain (Fig. 5A). Thus, we followed the interaction of antibodies targeting the NCT ECD and NCT CT (cytoplasmic domain) by fluorescence resonance energy transfer (FRET). Increased FRET efficiency in HEK293 cells treated with 1 µM inhX, compared with that of non-treated cells, confirmed that the NCT ECD approaches the membrane core (Fig. 5B).

**Fig. 6.**
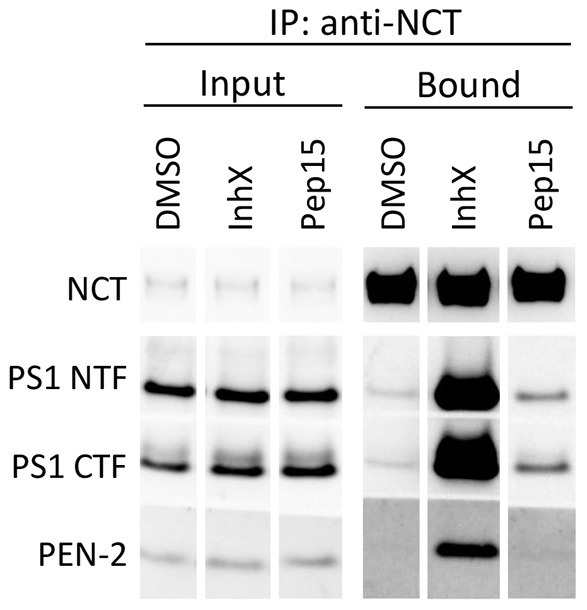
**InhX and pep15 treatments differentially increase the association of γ-secretase subunits in HEK293 cells.** Co-IP of γ-secretase subunits from HEK293 cells using an anti-NCT nanobody (Nb28, Fig. 1). Immunoprecipitation (IP) was performed in TX-100 buffer following overnight incubation with either 1 µM inhX (lanes 2 and 5) or 10 µM pep15 (lanes 3 and 6). InhX treatment stabilized the γ-secretase complex against dissociation in TX-100, whereas pep15 induced partial stabilization compared with samples treated with DMSO, supporting the electron microscopy results.

We wondered whether the increased compactness of γ-secretase that was observed in the presence of these two inhibitors of very different classes would affect the stability of the complex. We therefore performed experiments involving co-immunoprecipitation (coIP) of endogenous γ-secretase, using a nanobody against the NCT ECD (Nb28) (Fig. 1E,F) in CHAPSO, which keeps the complex together, or TX-100, which normally dissociates the complex (Fig. 6, lane 4). In agreement with Barthet et al. (2011) (Barthet et al., 2011) we found that γ-secretase is stable in the presence of inhX and partially stable in pep15 in TX-100 (Fig. 6, lanes 5 and 6). Thus, remodeling of the conformational landscape of γ-secretase upon inhibitor binding is observed in electron microscopy and translates into γ-secretase stabilization in TX-100.

### FAD mutation remodels the γ-secretase conformational landscape by enriching for an extended conformation

**Fig. 7.**
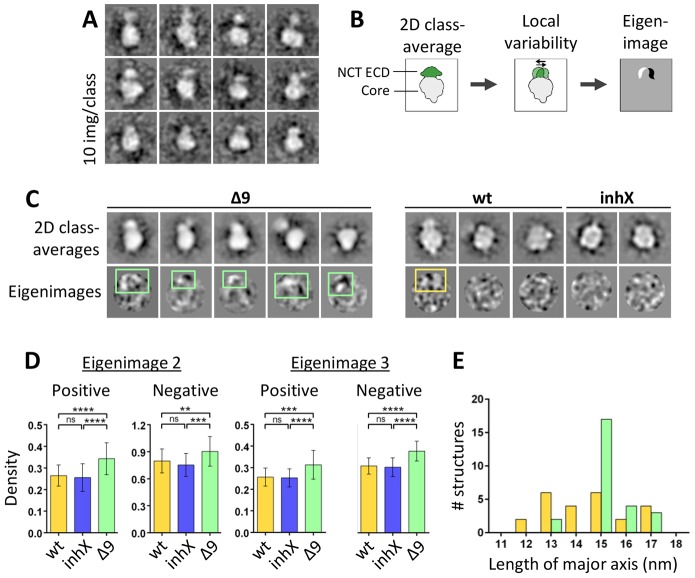
**The conformational landscape of γ-secretase is altered by PS1-Δ9 mutation.** (A) Class averages comprising a small number of images (∼10) per class, showing the expected membrane core and NCT ECD domains as observed in the wild type. The position of the NCT ECD varies considerably. (B) Illustration of the technique for identification of local variability in 2D classes: datasets were sorted into classes of similar features (represented in 2D class averages). Then, in order to assess local variability within classes, the images within each class were subjected to MSA and the resulting eigenimages were examined. Strong positive and negative density peaks in eigenimages indicate local variability. (C) Representative class averages from the γ-secretase-Δ9 dataset (left panel, first row) and corresponding eigenimage analysis of the images within classes (left panel, second row). Green squares mark the position of significant positive and negative density peaks in eigenimages, indicating systematic local variation at these positions. The same analysis was performed for the class averages of the wild-type (wt) γ-secretase and γ-secretase–inhX (right panel), resulting in significantly weaker density peaks in eigenimages, although some peaks were observed in the extended conformation class averages (yellow square). (D) Quantification of local variability in the wild-type γ-secretase (yellow), γ-secretase–inhX (blue) and γ-secretase-Δ9 (green). Reference-free aligned images from the three datasets were classified into 30 classes and eigenimages were calculated within each class. The density of the highest and lowest peaks was measured in eigenimages 2 and 3 (eigenimage 1 represents the total average). γ-secretase-Δ9 shows significantly stronger density in peaks compared with wild-type γ-secretase and γ-secretase–inhX, indicating local variations. Data show the mean±s.d.; **P<0.01; ***P<0.001; ****P<0.0001; ns, non-significant. (E) Length distribution of the different γ-secretase-Δ9 structures (supplementary material Fig. S3E–G) based on measurement along their major axis.

We wondered what the effect of a FAD mutation would be on the conformational landscape of the complex. The FAD mutant PS1 delta exon 9 (Δ9) lacks part of the extracellular loop between PS1-CTF and -NTF and is therefore expected to provide visible alterations to the complex. It should be noted that, similar to other FAD mutations, Δ9 results in an altered profile of Aβ production (Chávez-Gutiérrez et al., 2012; Perez-Tur et al., 1995). We expressed and purified the FAD Δ9 γ-secretase complex, and collected and processed electron microscopy images as indicated above. Windowed particles and class averages showed the same features as wild-type γ-secretase, indicating that the Δ9 complex grossly maintains the features of wild-type γ-secretase (Fig. 7A). The NCT ECD was visible as a separate density in more than a third of the class averages and appeared to be remarkably mobile, adopting different shapes and variable positions with respect to the membrane core (Fig. 7A,B). This made 3D reconstruction very difficult and, therefore, we limited our investigation by analyzing the conformational variability in γ-secretase Δ9 using 2D eigenimage analysis (Fig. 7B–D) (Elad et al., 2008; Elad et al., 2007). Using this approach, the global variability in the dataset is reduced by classifying the particles first based on their overall shape, allowing the identification of more subtle local variability within classes as density peaks in eigenimages. Interestingly, the occurrence of significant positive and negative density peaks indicates systematic structure variation in the region of the NCT ECD, even in class averages that contained no obvious density for the NCT ECD (owing to high domain flexibility) (Fig. 7C, Δ9 last image). Wild-type γ-secretase and inhX-bound γ-secretase datasets showed significantly weaker density peaks in eigenimage, although some variation in the NCT ECD region was observed in extended-conformation classes of wild-type γ-secretase (Fig. 7C, yellow square). Finally, we confirmed an enrichment of intermediate to extended conformations due to a highly mobile NCT ECD, as seen in the length of the major axis (Fig. 7E; supplementary material Fig. S3E–G).

## DISCUSSION

The ‘ensemble model’ for allostery states that the diversity and distribution of conformations present in solution underlies enzyme activity (Boehr et al., 2009; Motlagh et al., 2014). In this view, the conformational landscape, rather than specific ‘active’ or ‘inactive’ conformations, provides the basis for the biochemical function of proteins. The relevance of this hypothesis has been demonstrated in structural studies, for example of the regulation of G-protein-coupled receptors (GPCRs) (Nygaard et al., 2013), the ribosome (Fischer et al., 2010), the transcription factor TFIID (Cianfrocco et al., 2013) or the integrins (Takagi et al., 2002).

**Fig. 8.**
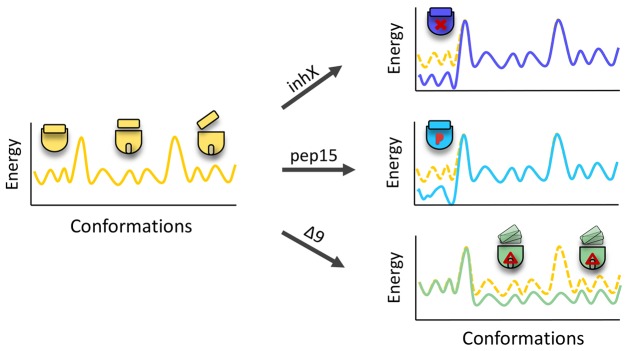
**Energy landscape view of γ-secretase.** Schematic energy landscape view of the conformational diversity in wild-type γ-secretase and its rearrangement in γ-secretase–inhX, γ-secretase–pep15 and γ-secretase-Δ9 populations. Wild-type γ-secretase fluctuates between three major conformations. InhX and pep15 differentially stabilize the most compact conformation observed in the untreated γ-secretase, whereas the Δ9 mutation increases conformational flexibility and enriches the subpopulations with intermediate to extended conformations. The overall conformational landscape is multidimensional; however, here it is reduced to one dimension, concentrating on the length of the major axis.

We present here a first characterization of γ-secretase structure dynamics. The most remarkable conclusion from our work is that γ-secretase exists as an ensemble of conformers (Fig. 8). The equilibrium between conformers is modulated by inhibitor binding to the active or substrate-binding sites, providing functional relevance to this observation. It is also interesting that a mutation causing Alzheimer disease increases the conformational heterogeneity of the complex. Therefore, we suggest that clinical mutations in presenilin might cause an overall destabilization of the structure, leading to increased flexibility. We speculate that such destabilization could result in destabilization of the enzyme–substrate state and promote product release, providing an explanation for the higher release of longer Aβ peptides from the complex in the presence of FAD mutations (Chávez-Gutiérrez et al., 2012).

Importantly, independent biochemical experiments show that inhX and pep15 binding, which resulted in increased compactness of γ-secretase in the electron microscopy images, dramatically affected the biophysical stability of the complex in a dissociating detergent (TX-100). In addition, our FLIM data supports the inference that such large conformational shifts of the NCT ECD happen in intact cells. In agreement, the NCT ECD is crucial for the assembly, maturation and stability of γ-secretase (Chávez-Gutiérrez et al., 2008). Furthermore, several publications using FLIM showed how γ-secretase undergoes conformational changes in the intact cell membrane (Berezovska et al., 2005; Li et al., 2014; Lleó et al., 2004; Shelton et al., 2009; Uemura et al., 2010). Thus, molecular interactions affect γ-secretase function by changing the frequencies of subpopulations in the conformational landscape. Allosteric behavior, substrate affinity and catalytic mechanisms are therefore determined by the ensemble average. Thus, the observed conformational landscape truly reflects the inherent dynamics of γ-secretase.

The observed dynamics have particular implications for future structural and functional studies. Structural homogeneity is determinant for protein crystallization and the generation of high-resolution data by electron microscopy. It should be noted that the recently published high-resolution structure (Lu et al., 2014) is based on a careful selection of ∼5–10% of the particles from the purified material. We suggest that such ‘in silico’ purification and the use of amphipol masked potential structural heterogeneity, enabling the high-resolution model to be built. The observed heterogeneity might also explain why different γ-secretase structures published previously (Lazarov et al., 2006; Li et al., 2014; Ogura et al., 2006; Osenkowski et al., 2009; Renzi et al., 2011) are rather divergent. The current study suggests that pre-incubation with inhX might help to reduce heterogeneity. In addition, other ligands (e.g. nanobodies) that stabilize the protease complex in particular conformations might also help studies aiming to elucidate the atomic structure of the complex.

On the functional side, the conformational fluctuations observed in the present study indicate that a more complete description of the γ-secretase catalysis has to take into account the interconverting conformers that might exist at each step of the catalytic reaction. The conformational fluctuations give rise to ensembles of Michaelis–Menten enzyme–substrate and enzyme–product complexes and their corresponding transition states, and these ensembles will undergo equilibrium shifts depending on the environment. In fact, the concept that multiple enzyme conformers catalyze reactions in parallel is studied by ‘catalytic networks’ (Benkovic et al., 2008). The incorporation of dynamics into the studies of γ-secretase intramembrane proteolysis is thus crucial for understanding its function and elucidating its structure at high resolution.

## MATERIALS AND METHODS

### Antibodies

The following antibodies were used for labeling: affinity-purified rabbit polyclonal anti-APH-1a (B80.3) and affinity-purified monoclonal anti-NCT-CT 9C3 (Annaert et al., 2001; Esselens et al., 2004); affinity-purified rabbit polyclonal against N-terminal amino acids 103–124 of NCT (anti-NCT-ECD, ab24741) and affinity-purified rabbit polyclonal against 13 amino acids at the C-terminus of PEN-2 (anti-PEN-2, ab62514) (both from Abcam); rabbit polyclonal against C-terminal amino acids 450–467 of PS-1 (anti-PS1-CT, S182; Sigma). The following antibodies were used for detection by western blotting: rabbit anti-PEN-2 (B126.1; Annaert et al., 2001; Esselens et al., 2004); anti-human-PS1-loop (MAB5232, Chemicon); mouse anti-NCT (BD Transduction Laboratories); rat anti-PS1-NTF (MAB1563, Millipore); and anti-FLAG M2 (Sigma).

### Immunoprecipitation with nanobodies

Hi5 cells overexpressing the γ-secretase complex were lysed in either 1% CHAPSO or 1% Triton X-100 for 1 h on ice. Insoluble material was removed by ultracentrifugation for 45 min at 100,000 ***g***. A total of 100 µg of solubilized material protein was incubated with the indicated nanobodies, which were previously covalently coupled to NHS-beads (GE Healthcare) according to the manufacturer's instructions. 25 µl of nanobody beads (0.3 mg of nanobodies per ml of beads) were used per condition. Immunoprecipitations were carried out in PBS buffer containing 1% CHAPSO or 1% Triton X-100 in a total volume of 250 µl, overnight at 4°C. Bound and unbound fractions were analyzed by SDS-PAGE and western blotting.

### Overexpression and purification of wild-type and Δ9 γ-secretase

The γ-secretase complex was overexpressed in Hi5 insect cells from a single vector carrying the four subunits. NCT was expressed as a GFP fusion. Purification followed three steps (Fig. 1B,C). Expression and first-step purification using an anti-GFP nanobody were performed as described recently (Acx et al., 2014). A second immunoaffinity step, performed with a His-tagged conformational nanobody (Nb30) that recognizes the γ-secretase complex and not the individual subunits (Fig. 1E,F), enriched for mature and active protease complexes. The Nb30–γ-secretase complex was purified by immobilized metal ion affinity chromatography (IMAC) and eluted with 250 mM imidazole. Finally, gel filtration in a Superose6 column (GE Healthcare) removed unbound nanobody and exchanged detergent for 0.1% LMNG. Purified γ-secretase complexes were tested in an *in vitro* activity assay as described previously (Acx et al., 2014).

### FLIM-FRET

HEK293 cells were seeded at 150,000 cells per well on glass coverslips in 12-well plates. After 24 h, medium was refreshed and 1 µM of L-685,458 (inhX, Calbiochem) or DMSO was added. After overnight incubation, cells were fixed in 4% paraformaldehyde for 10 min, blocked (2% BSA, 2% FBS and 1% gelatin in PBS supplemented with 5% serum) and incubated with primary antibodies (anti-NCT-ECD, ab24741; anti-NCT-CT, 9C3) overnight at 4°C. Cells were subsequently incubated with Alexa-Fluor-488- or Alexa-Fluor-555-conjugated secondary antibodies for 1 h at room temperature, washed with TBS and mounted in Vectashield H-1000 (Vector Labs).

The relative proximity between fluorophore-labeled NCT ECD and CT was assessed by the previously validated fluorescence lifetime imaging microscopy (FLIM) analysis as described elsewhere (Berezovska et al., 2005; Uemura et al., 2010). Briefly, the baseline lifetime (*t*1) of the Alexa Fluor 488 fluorophore (negative control, FRET absent) was measured in the absence of an acceptor fluorophore. Upon excitation of the donor fluorophore in the presence of Alexa Fluor 555 acceptor fluorophore, the donor lifetime shortens (*t*2) owing to FRET if the donor and acceptor are less than 5–10 nm apart. The degree of donor fluorophore lifetime shortening (FRET efficiency) correlates with the change in proximity between the two fluorescently labeled domains of the NCT molecule. The percent FRET efficiency is calculated using the following equation: %E_FRET_ = 100*(*t*1−*t*2)/*t*1. A mode-locked Chameleon Ti:Sapphire laser (Coherent Inc., Santa Clara, CA) sending a femtosecond pulse every ∼12.5 nsec was used to excite the donor fluorophore (two-photon excitation at 800-nm wavelength). Donor fluorophore lifetimes were recorded using a high-speed photomultiplier tube (MCP R3809; Hamamatsu, Bridgewater, NJ) and a fast time-correlated single-photon counting acquisition board (SPC- 830; Becker & Hickl, Berlin, Germany). SPCImage software (Becker&Hickl, Berlin, Germany) was used to determine the donor fluorophore lifetimes by fitting the data to one (negative control) or two (acceptor present) exponential decay curves.

### Sample preparation for electron microscopy

Purified γ-secretase sample was diluted 1∶10 to 0.035 mg/ml in buffer without detergent (25 mM PIPES pH 7.4, 150 mM NaCl, 5% glycerol) immediately prior to fixation on the electron microscopy grid. We diluted the detergent in order to reduce background noise in electron microscopy micrographs. γ-secretase was stable and active at the final concentration of LMNG (0.01%), which is 10× the critical micelle concentration (Chae et al., 2010). Carbon-coated 400 mesh grids were glow-discharged and then incubated for 1 min with a 3-µl drop of 0.1 mg/ml inert nanobody, which has no affinity to γ-secretase and no effect on the structural analysis owing to its small size (15 kDa). We found that this pretreatment of the carbon surface improved staining quality. Then, 3 µl of purified sample was applied and negatively stained with 2% uranyl acetate. For preparation of the γ-secretase–inhX and γ-secretase–pep15 complexes, purified sample was diluted in buffer containing 10 µM of inhX or 1 µM pep15, respectively, and incubated for 1 h at room temperature. Antibody labeling for electron microscopy imaging was performed during the second step of the purification while γ-secretase was bound on the Nb30 beads. Beads with bound γ-secretase were incubated overnight at 4°C with the specified antibodies followed by washing and elution with 250 mM imidazole.

### Electron microscopy data collection and 2D and 3D image processing

Wild-type γ-secretase, γ-secretase–pep15 tilt pair (0° and 55°) and antibody-labeled γ-secretase datasets were collected on a Gatan US4000 CCD camera at 47,000× magnification (1.8 Å/pixel) using a Titan Krios (FEI, Eindhoven) operated at 200 kV and controlled using SerialEM (Mastronarde, 2005). The γ-secretase–inhX dataset was collected on a TVIPS 4k×4k CCD camera at 83,333× magnification (1.8 Å/pixel), using a Phillips CM120 Biotwin Ice operating at 120 kV. The γ-secretase Δ9 dataset and γ-secretase–inhX tilt pairs (0° and 25°, for tilt-pair validation) were collected on a TVIPS F224HD 2k×2k CCD camera (Gauting, Germany) at 113,744× magnification (2.1 Å/pixel) using a FEI T12 operating at 120 kV. Imaging was performed using a defocus range of 0.7–1.3 µm and an electron dose of 30 e^−^/Å^2^. The contrast transfer function was determined for each micrograph using CTFFIND3 (Mindell and Grigorieff, 2003). Particles from micrographs of all samples except antibody-labeled datasets were selected interactively using EMAN2 (Tang et al., 2007). Particles of the antibody-labeled datasets were selected automatically using a template-matching algorithm implemented in SPIDER (Frank et al., 1996; Rath and Frank, 2004). Particles were extracted to yield boxes of 256×256 pixels, and phase-corrected using SPIDER. After contrast transfer function correction, the box size was cropped to 160×160 pixels, images were binned by a factor of 2 and band-pass filtered between 25 Å and 200 Å. Filtered particles were subjected to ten iterations of reference-free alignment and classification using IMAGIC (van Heel et al., 1996), starting with classes of 20 images and gradually increasing the number of images per class to ∼500, depending on sample homogeneity.

For random conical tilt (RCT) reconstruction of the wild-type γ-secretase, 9,774 tilt pairs were selected interactively using EMAN2. The 0° tilt particles were subjected to reference-free alignment and classification, resulting in 30 classes. A total of 30 3D RCT maps were reconstructed from the corresponding tilted particles, and each 3D map was refined internally against its tilted particles using IMAGIC. Initial RCT structures were then refined iteratively with decreasing angular increments against a dataset of 36,093 0° tilt particles using SPIDER. A cross-correlation threshold was applied every round to select the best-matching images for each structure. Additionally, in order to minimize the effect of overpopulated orientations on the angular distribution, the number of images per orientation was limited for 3D reconstruction. In total, 6000–8000 molecular views (∼20% of the dataset) were included in each structure. The Fourier shell correlations (FSCs) from ‘gold standard’ refinement half-sets (Scheres and Chen, 2012) indicate resolutions from 23 to 25 Å at FSC = 0.143 for wild-type structures (supplementary material Fig. S2A,C,E). Structures that refined until convergence, showed the common features as described in the results above and displayed good match between class averages and re-projections were subsequently aligned in three dimensions and subjected to 3D classification. Most of the complexes featured preferred orientation on the carbon support. Therefore, structures could be roughly aligned by manual rotation about the axis perpendicular to the carbon support. Manual alignment was followed by four iterations of automated 3D alignment using UCSF Chimera (Pettersen et al., 2004), in which a 3D global average was calculated and individual structures were aligned to the global average structure. Structures showing poor cross-correlation were discarded during the process. Aligned 3D structures were subjected to multivariate statistical analysis (MSA)-based hierarchical ascendant classification using IMAGIC. Unsupervised 3D classification in RELION (Scheres, 2012) was performed using the 3D global average, filtered to 80 Å, as an initial model. The low-pass filtered data were classified into ten 3D classes, which showed the same architecture and heterogeneity as above, therefore independently confirming the results.

The γ-secretase–inhX and γ-secretase–Δ9 datasets contained 9267 and 11,443 particles, respectively. The 30 initial RCT models from the wild-type data (filtered to 50 Å) were used as starting models for 3D reconstruction of both datasets. We used the same refinement procedure as for the wild type, except that ∼3000–4000 images were included in each structure. A total of 22 and 25 of the γ-secretase–inhX and γ-secretase-Δ9, respectively, were aligned and classified as described above for the wild-type γ-secretase structures. Structures of all datasets were aligned to the same coordinates.

The γ-secretase–pep15 dataset was collected and processed as described for wild-type γ-secretase above. 6777 tilt pairs were selected interactively and classified into 15 classes using reference-free alignment, which were then used to reconstruct 15 3D maps using the RCT method. 13 maps were used for 3D alignment and classification.

Antibody-labeled datasets contained 10,000 particles each. Datasets were processed separately by reference-free alignment and classification as described above, followed by classification into classes of 20 images. Class averages of poor quality were discarded, and the remaining class averages were aligned to class averages of the wild-type γ-secretase (non-labeled) dataset using SPIDER. We then selected class averages from the labeled dataset showing the same γ-secretase features as in the matching class average of the non-labeled dataset, and containing an extra density at the periphery. Only extra density appearing consistently at the same position relative to γ-secretase density was interpreted as bound antibody.

The tilt-pair test (Rosenthal and Henderson, 2003) for validation of the overall correctness of the inhX-treated structure was performed on 46 particles taken from a single micrograph using the EMAN2 e2tiltvalidate procedure.

Fitting of the high resolution map as a rigid body (Lu et al., 2014) was performed automatically using UCSF Chimera (Pettersen et al., 2004), followed by small manual adjustments of the NCT ECD atomic coordinates position.

## Supplementary Material

Supplementary Material
