# Supplementary material for: The dynamic conformational landscape of γ-secretase
Source: J Cell Sci. 2015 Feb 1;128(3):589–98. doi: 10.1242/jcs.164384 (PMC4311135; doi:10.1242/jcs.164384)
Supplement: Supplementary Material [file supp_128_3_589__index.html]

The dynamic conformational landscape of γ-secretase — Supplementary Material 

# The dynamic conformational landscape of γ-secretase

## JCS164384 Supplementary Material

**Files in this Data Supplement:**

- **Supplementary Material**
