# Supplementary material for: The dynamic conformational landscape of γ-secretase
Source: J Cell Sci. 2015 Feb 1;128(3):589–98. doi: 10.1242/jcs.164384 (PMC4311135; doi:10.1242/jcs.164384)
Supplement: Supplementary Material [file supp_128.3.589_JCS164384.pdf]

Figure S1

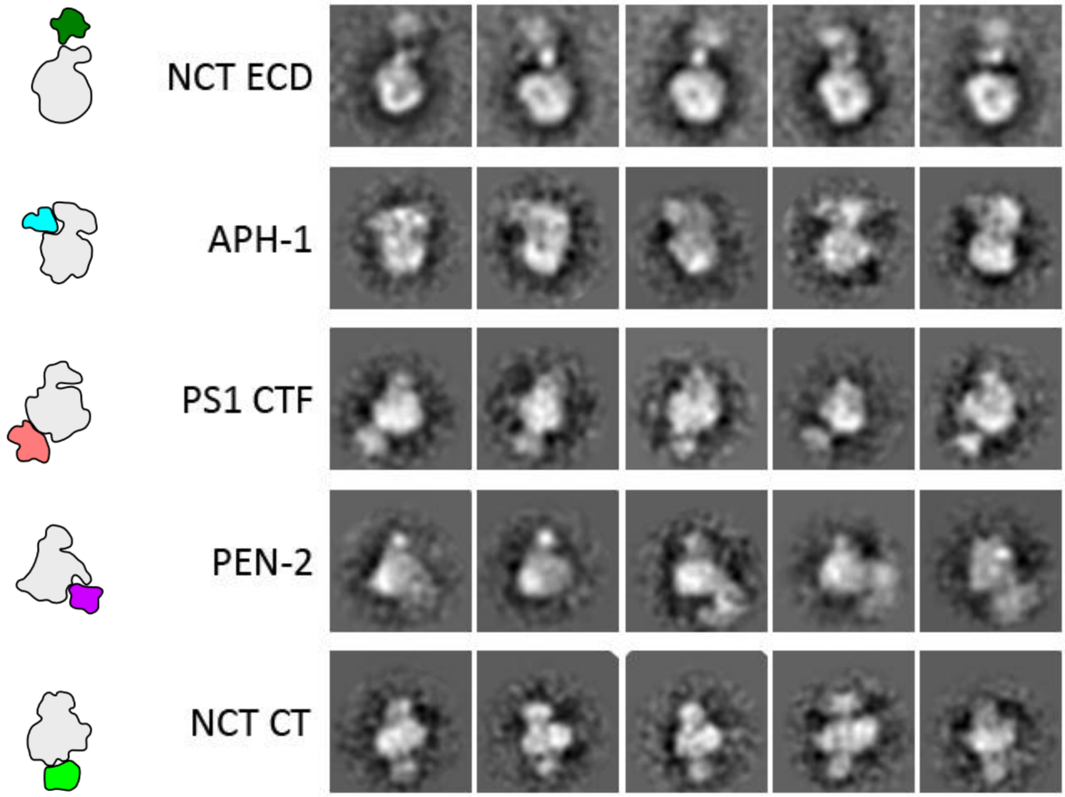

Antibody labeling in 2D class averages as presented in Fig. 2F-H. For each labeled subunit, the outline (first column) and class average (second column) from Fig. 2H and G respectively are presented along with 4 additional class averages, showing antibody density at the same position with respect to the γ-secretase.

Figure S2

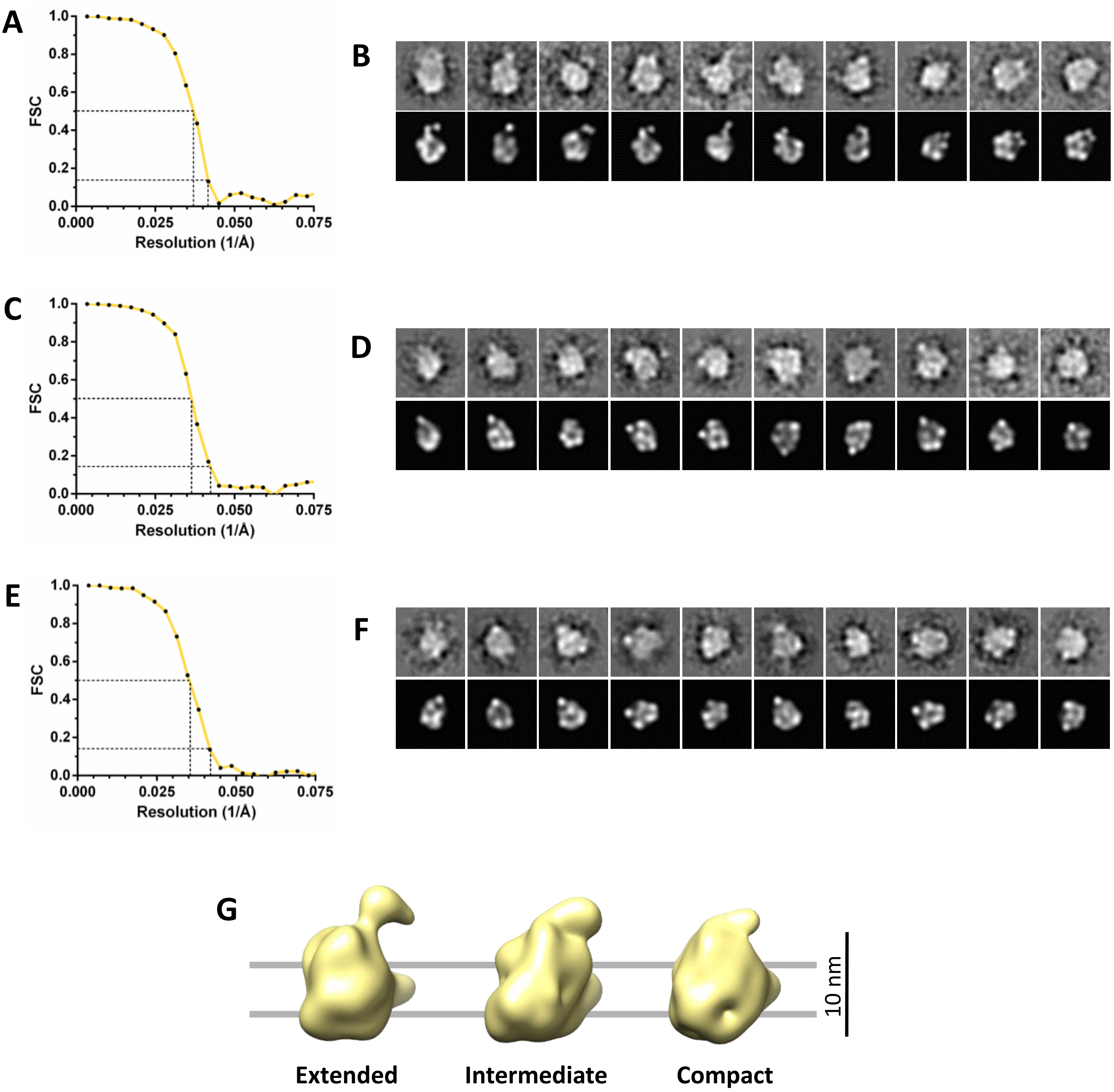

(A, C, E) FSCs using ‘gold standard’ (Scheres and Chen 2010) generated for the extended, intermediate and compact wt γ-secretase structures in Fig. 3B-D respectively. Dotted lines in the FSC curves are 0.5 and 0.143 cutoffs.

(B, D, F) Class averages and matching re-projections for each structure.

(G) 3D reconstruction and unsupervised 3D classification performed independently using RELION (Scheres, 2012)

Figure S3

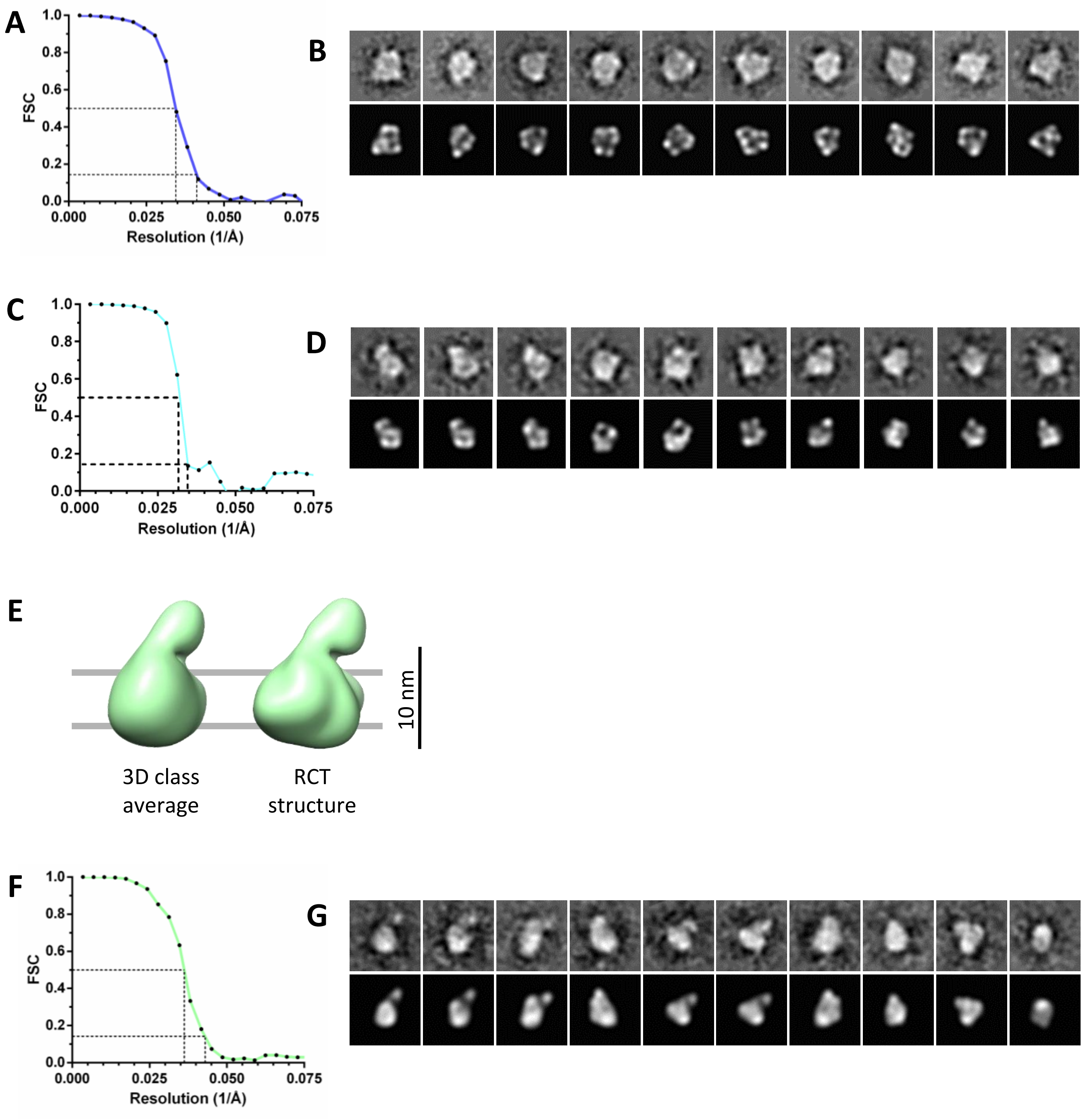

FSCs from ‘gold standard’ refinement of half data sets (Scheres and Chen 2010), class averages and matching re-projections shown for the representative structures of the γ-secretase-inhX (A,B) γ-secretase-pep15 (C,D) γ-secretase-Δ9 (F,G).

(E) 3D average and representative RCT structure from the γ-secretase-Δ9 data set.

Figure S4

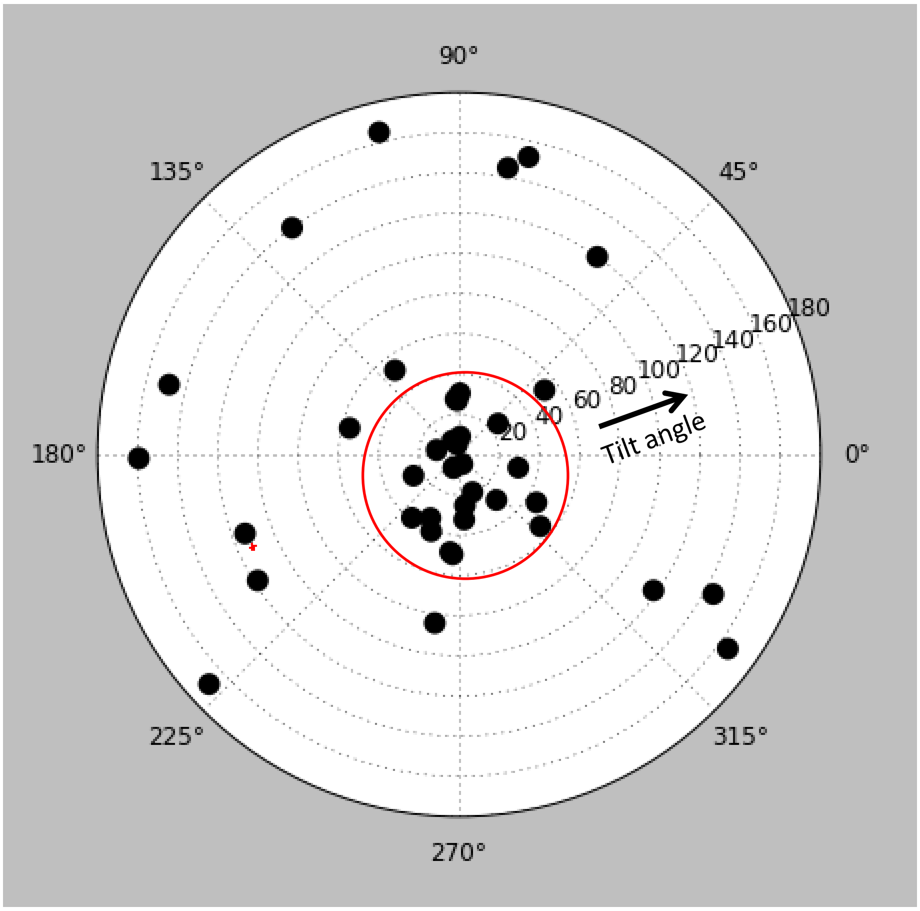

Tilt-pair validation plot (Roenthal and Henderson, 2003) for the γ-secretase-inhX structure. Micrograph was collected at 0° and 25°. The average tilt angle of the clustered tilt-pairs surrounded by a red circle is 24.8°, confirming the overall correctness of the structure.
